# Stearic acid-modified PSMA-targeting peptide–drug conjugate for long-acting prostate cancer therapy

**DOI:** 10.1039/d5sc08259e

**Published:** 2026-03-05

**Authors:** Ziwen Qiu, Xiaorui Zheng, Shoumei Pan, Yingtao Zhong, Xiayun Chen, Xuejun Wen, Xin Chen, Shiying Li, Hong Cheng, Xiaoyuan Chen

**Affiliations:** a Department of Pulmonary and Critical Care Medicine, Zhujiang Hospital, Southern Medical University Guangzhou 510280 China chen_xin1020@163.com; b Biomaterials Research Center, School of Biomedical Engineering, Southern Medical University Guangzhou 510515 China chengh@smu.edu.cn; c Guangdong Provincial Key Laboratory of Molecular Target & Clinical Pharmacology, The NMPA, State Key Laboratory of Respiratory Disease, The School of Pharmaceutical Sciences, Guangzhou Medical University Guangzhou 511436 China lisy-sci@gzhmu.edu.cn; d Department of Diagnostic Radiology, Yong Loo Lin School of Medicine, National University of Singapore 119074 Singapore; e Shandong Provincial Key Laboratory of Precision Oncology, Shandong Cancer Hospital and Institute, Shandong First Medical University, Shandong Academy of Medical Sciences Jinan 250117 China chen9647@gmail.com

## Abstract

Prostate-specific membrane antigen (PSMA)-targeting peptide–drug conjugates (PDCs) offer promise for the treatment of PSMA-positive prostate cancer, but their applications are limited by rapid clearance, poor pharmacokinetics, and low efficacy. Herein, we design a series of PDCs incorporating rational functional moieties, including a Glu–Ureido–Lys PSMA-targeting ligand, the cytotoxic payload monomethyl auristatin E (MMAE), and a cathepsin B-cleavable Val–Cit linker, combined with structural modifications such as free amine exposure, acetylation, stearic acid acylation, or *p*-iodophenylbutyric acid (PIBA) conjugation. Among them, the stearic acid-modified PSMA-targeting PDC (PDC-C18) is identified as a long-acting candidate for treating prostate cancer. PDC-C18 rapidly forms stable nanocomplexes with human serum albumin (HSA) through hydrophobic interactions, effectively shielding its hydrophobic moiety while preserving PSMA specificity. Notably, PDC-C18 exhibits a more than 160-fold extension in half-life compared to conventional PDCs, prolongs mean residence time (MRT) from 12.47 h to 23.93 h relative to PIBA-modified PDCs, and achieves a 60-fold reduction in clearance rate. This long-acting property translates into a remarkable tumor suppression rate of 96.91%, simultaneously avoiding the side effects associated with traditional PIBA modification strategies. Overall, this study presents an effective approach to improving the pharmacokinetic behavior of PDCs and provides valuable insights for the development of next-generation tumor-targeted therapies.

## Abstract 2

Prostate-specific membrane antigen (PSMA)-targeting peptide–drug conjugates (PDCs) offer promise for the treatment of PSMA-positive prostate cancer, but their applications are limited by rapid clearance, poor pharmacokinetics, and low efficacy.

## Graphical abstract

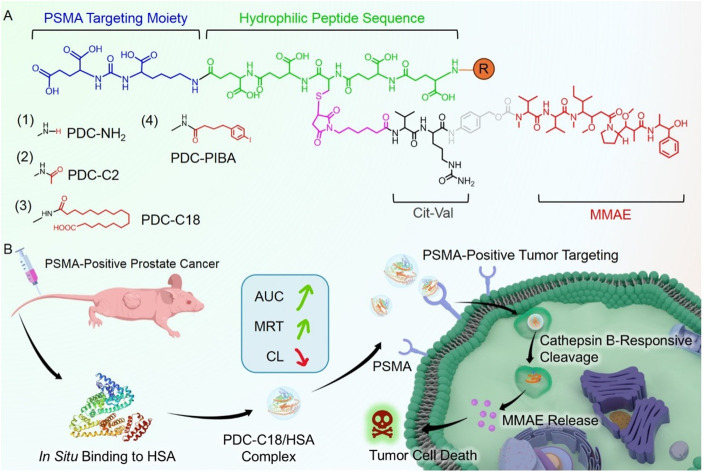

## Introduction

Prostate cancer is one of the most prevalent malignancies in men worldwide, yet effective treatment options remain limited.^1^ The development of new therapies is therefore critical for improving patient prognosis. Among emerging strategies, antibody-drug conjugates (ADCs) have gained considerable attention by combining antigen-specific targeting with potent cytotoxic payloads.^2^ For example, a humanized anti-PSMA IgG1 monoclonal antibody conjugated with monomethyl auristatin E (MMAE) has been demonstrated to selectively eliminate prostate cancer cells expressing prostate-specific membrane antigen (PSMA) through targeted delivery of microtubule inhibitors.^3^ However, despite encouraging preclinical and clinical results, no PSMA-targeting ADC has yet been approved by the U.S. Food and Drug Administration (FDA) for prostate cancer treatment, owing to inherent limitations such as large molecular size resulting in poor tumor penetration, high production costs, and potential immunogenicity.^4^

Peptide–drug conjugates (PDCs) have emerged as an attractive alternative.^5,6^ Compared with ADCs, PDCs possess a substantially smaller molecular size, which facilitates deeper tissue penetration, particularly in poorly permeable solid tumors. Additionally, peptides generally have lower immunogenicity than antibodies, thereby reducing the risk of immune-related side effects and improving suitability for repeated administration in receptor-targeted precision therapy. Compared to AptDCs, PDCs overcome the instability of nucleic acid-based ligands. While RNA aptamers offer high specificity, they are prone to nuclease degradation and often display circulation half-lives without extensive chemical modifications, limiting their effectiveness *in vivo*. In contrast, peptide-based ligands offer superior chemical stability and greater flexibility for structural optimization.^7^ Their clinical potential is underscored by peptide-based drugs already in use, such as ^177^Lu-dotatate for treating somatostatin receptor-positive gastroenteropancreatic neuroendocrine tumors, and PSMA-targeting radiopharmaceuticals (^68^Ga-PSMA-11 and ^18^F-DCFPyL) approved for prostate cancer imaging.^8^ In particular, the glutamate–urea–lysine (Glu–Ureido–Lys) scaffold has been demonstrated to be a high-affinity ligand for PSMA, providing a solid foundation for therapeutic PDC design.^9^ Nevertheless, the clinical translation of PDCs has been hindered by unfavorable pharmacokinetics in rapid renal clearance, short circulation half-lives, and diminished therapeutic efficacy.^10^

Conventional strategies such as PEGylation and glycosylation have been widely employed to prolong drug half-life, reduce clearance, and enhance stability, yet these modifications often cause drawbacks such as potential immunogenicity and interference with receptor binding.^11^ A promising alternative is to exploit human serum albumin (HSA)-the most abundant plasma protein in circulation-as a natural carrier.^12^ With a half-life of 19 days and multiple ligand-binding pockets, HSA can reversibly associate with therapeutics to prolong systemic exposure, reduce renal clearance, and enhance pharmacological effects.^13^ To leverage this property, various noncovalent strategies have been developed, including hydrophobic interactions with Evans Blue, ibuprofen, or 4-(*p*-iodophenyl)butyric acid (PIBA), all of which can extend circulation time and improve bioavailability.^14^ Notably, fatty acid modification has been clinically validated as a particularly effective strategy. For example, semaglutide achieves strong HSA binding through fatty acid acylation, markedly extending its half-life and enabling once-weekly dosing.^15^ This precedent highlights the translational potential of fatty acid modification in improving the pharmacokinetics of emerging therapeutic modalities.^5,16^ However, fatty acid-modified PDCs remain largely underexplored. While fatty acid acylation has been well-established for peptide hormones and biologics, its application in PDCs has received little attention. Systematic investigation is therefore warranted to determine whether fatty acid modification can effectively overcome rapid renal clearance and translate into meaningful improvements in antitumor efficacy.

**Scheme 1:**
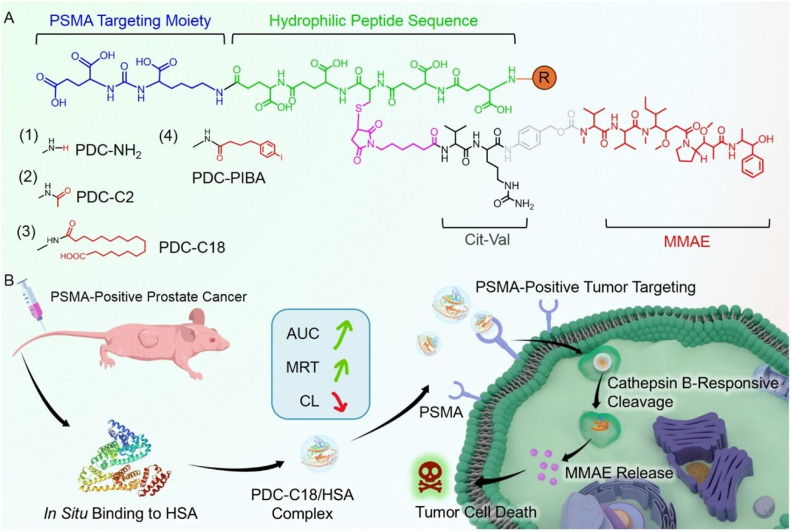
Design and mechanism of a PSMA-targeting peptide–drug conjugates (PDCs) bearing albumin-binding moieties. (A) The PDCs consist of a Glu–Ureido–Lys ligand for PSMA recognition, a cathepsin B-cleavable Val–Cit linker, and the cytotoxic payload MMAE, with optional albumin-binding units (NH_2_, C2, C18, or PIBA) appended. (B) After intravenous administration, the PDCs bind *in situ* to human serum albumin (HSA), thereby prolonging systemic exposure (AUC) and mean residence time (MRT) while reducing clearance (CL). Subsequently, the PDCs are internalized into PSMA-positive prostate cancer cells *via* PSMA-mediated uptake, where cathepsin B in lysosomes cleaves the Val–Cit linker and triggers self-immolative release of MMAE, ultimately leading to effective tumor cell killing.

In this study, we developed a series of PSMA-targeting PDCs comprising a Glu–Ureido–Lys ligand for PSMA recognition, a cathepsin B-cleavable Val–Cit linker, and the cytotoxic payload MMAE (Scheme 1A). To enhance pharmacokinetics, we engineered albumin-binding variants-free-amine exposure, acetylation, stearic acid acylation, and PIBA conjugation-and systematically characterized their affinities for HSA and *in vivo* PK profiles. As shown in Scheme 1B, the stearic acid derivative PDC-C18 rapidly and stably associated with HSA *via* hydrophobic interactions, markedly increasing exposure (AUC) and mean residence time (MRT) while reducing clearance (CL). HSA engagement suppressed nonspecific uptake yet preserved PSMA-mediated internalization. Further, cathepsin B cleaved the Val–Cit linker to trigger self-immolative release of MMAE within lysosomes, yielding potent and selective cytotoxicity toward PSMA-positive prostate cancer cells. Compared to the PIBA-modified analogue (PDC-PIBA), PDC-C18 exhibited stronger antitumor efficacy and reduced hepatic and renal toxicity. Altogether, fatty acid-mediated albumin binding might provide a powerful strategy for optimizing the pharmacokinetics, safety, and therapeutic index of PDCs, positioning PDC-C18 as a promising long-acting candidate for the treatment of prostate cancer.

## Results and discussion

### Structural optimization and validation of binding affinity of PDCs to HSA

**Fig. 1:**
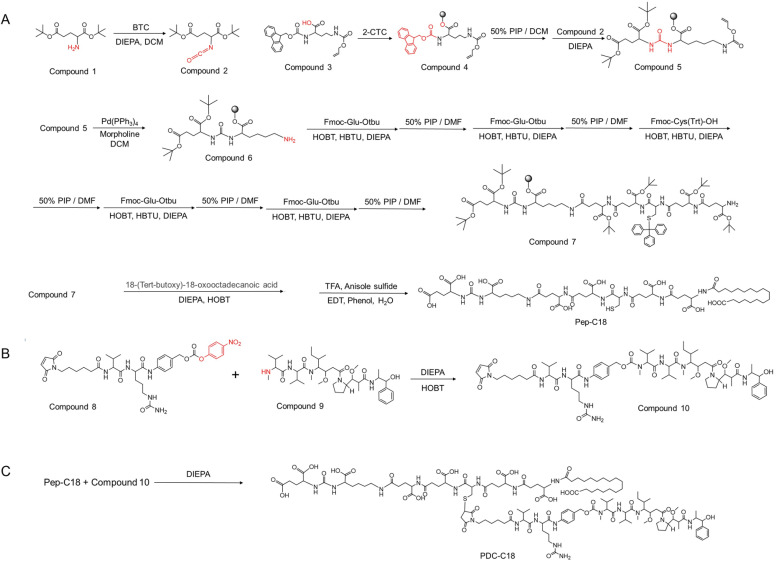
Synthetic routes to PDC-C18. (A) Synthesis of peptide-C18. (B) Synthesis of linker-MMAE. (C) Synthesis of PDC-C18.

**Fig. 2:**
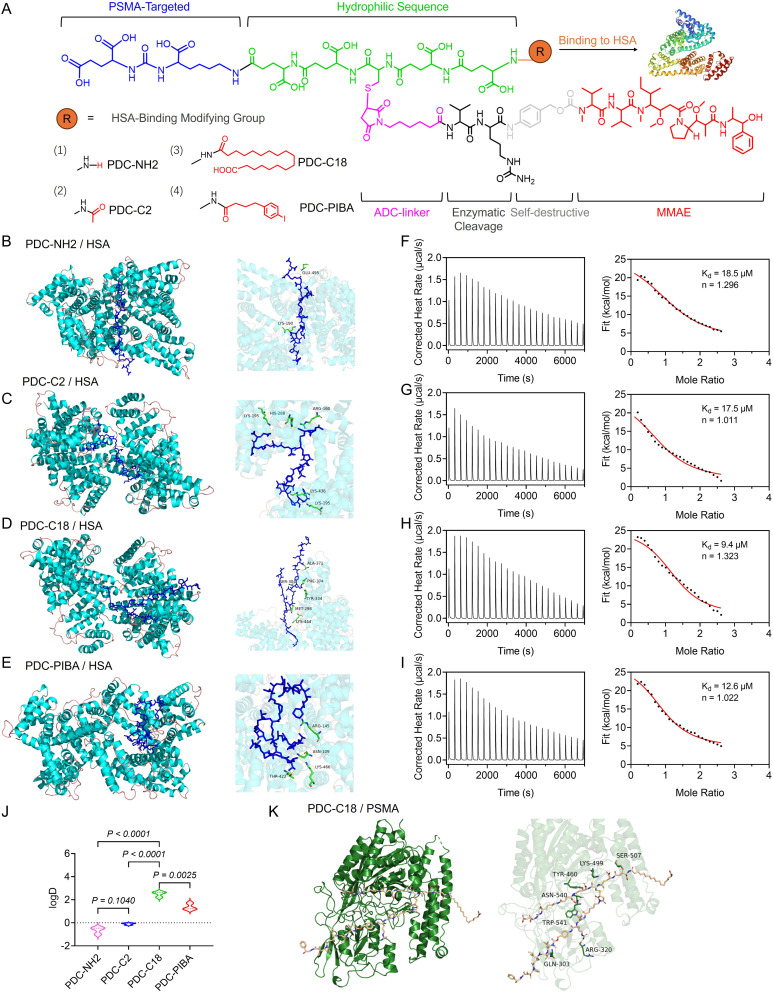
Structure optimization and HSA binding affinity of PDCs. (A) Chemical structure and HSA binding ligand optimization of PDCs. (B) Molecular docking simulations of (B) non-HSA binding PDC (PDC-NH_2_), (C) acetyl-modified PDC (PDC-MC2), (D) stearic acid-modified PDC (PDC-C18), and (E) *p*-iodophenylbutyric acid-modified PDC (PDC-PIBA). Thermogram curves and ITC fitting curves of HSA titrated into (F) PDC-NH_2_, (G) PDC-C2, (H) PDC-C18, and (I) PDC-PIBA. *K*_d_ represents the binding affinity constant, and *n* denotes the binding stoichiometry. (J) Partition coefficients (log *D*) of PDC-NH_2_, PDC-C2, PDC-C18, and PDC-PIBA. (K) Molecular docking simulation of PDC-C18 with PSMA protein. Data in violin plots represent the full distribution of individual data points, with median indicated. Statistical significance was determined using one-way ANOVA followed by Tukey's multiple comparisons test. *P* values are indicated in the figure.

Conventional PDCs are typically composed of three elements: a peptide-based targeting ligand, a cytotoxic payload, and a cleavable linker. However, their clinical translation has been largely constrained by unfavorable pharmacokinetics. To address this challenge, researchers have explored structural modification strategies, such as *p*-iodophenylbutyric acid (PIBA) conjugation, which is one of the most widely adopted methods to enhance drug half-life for improving HSA binding. Building on this rationale, we designed a series of PDCs that integrated rationally selected functional moieties, including a Glu–Ureido–Lys PSMA-targeting ligand, the potent cytotoxin MMAE, and a cathepsin B-cleavable Val–Cit linker (Fig. 1). In addition, as shown in Fig. 2A, systematic structural modifications were introduced: free amine exposure (PDC-NH_2_), acetylation (PDC-C2), stearic acid acylation (PDC-C18), and PIBA conjugation (PDC-PIBA). These PDCs were synthesized *via* standard solid-phase peptide synthesis and Michael addition reactions, and their structures were confirmed by mass spectrometry (Fig. S1–S9). Specifically, the hydrophilic segment was designed to improve aqueous solubility and mitigate excessive hydrophobicity that could otherwise induce aggregation or unfavorable biodistribution. In addition, the incorporation of a hydrophilic sequence contributed to improved circulation stability and bioavailability by balancing the overall amphiphilicity of the PDCs, particularly in the case of C18 modification. This balanced amphiphilicity helped maintain molecular dispersion in biological environments while supporting controlled payload release and effective *in vivo* delivery.

Molecular docking revealed distinct binding modes of the four PDCs with HSA (Fig. 2B–E). PDC-C18 anchored its stearate tail in a hydrophobic groove at the entrance of domain IIIA (Ala306, Ala371, Phe374, Lys372) while forming 14 hydrogen bonds and four salt bridges, yielding a strong hydrophobic–electrostatic lock. PDC-PIBA instead engaged 14 hydrogen bonds, one Lys-mediated salt bridge, and an I–N halogen bond with Lys432, but only two hydrophobic contacted, reflecting a more polar and directional binding mode. In contrast, PDC-NH_2_ and PDC-C2 exhibited fewer interactions and limited hydrophobic burial, consistent with weaker affinity. Site mapping further suggested that PDC-PIBA favored the IIA subdomain (Sudlow site I), whereas PDC-C18 predominantly occupied the hydrophobic entrance of domain IIIA, well suited for long alkyl chains. Thermodynamically, isothermal titration calorimetry (ITC) confirmed the docking trends, with PDC-C18 showing the strongest HSA affinity (*K*_d_ = 9.4 µM), followed by PDC-PIBA (12.6 µM), while PDC-C2 (17.5 µM) and PDC-NH_2_ (18.5 µM) bounded more weakly (Fig. 2F–I). All profiles fitted a 1 : 1 model (*n* ≈ 1.0–1.3), consistent with a dominant binding stoichiometry. Hydrophobicity measurements further supported these results: PDC-NH_2_ (log *D* = −0.64) and PDC-C2 (−0.10) were markedly less hydrophobic than PDC-C18 (2.50) and PDC-PIBA (1.37) (Fig. 2J). Together, these data indicated that greater hydrophobicity enhanced HSA association within this scaffold, with the stearoyl chain of C18 offering the most favorable balance of hydrophobic and polar interactions.

Finally, to evaluate whether stearic acid modification interfered with PDC recognition of PSMA, we quantified the interactions within the PDC-C18–PSMA complex (Fig. 2K). The central Glu–Ureido–Lys pharmacophore retained 13 hydrogen bonds in the active site, with geometries typical of strong H-bonds with H–A distances of 1.28–3.45 Å, D–A distances of 2.12–4.09 Å, and D–H⋯A angles of 105.8–167.2°. In addition, LYS499 formed a salt bridge (4.52 Å) with the ligand carboxylate, electrostatically anchoring the urea headgroup. By contrast, the C18 alkyl chain mainly made van der Waals contacts with the hydrophobic patch at the pocket entrance and resided in a solvent-exposed lateral groove, without penetrating or perturbing the hydrogen-bond/salt-bridge core that mediated recognition. The conformation in Fig. 2K also showed that the orientation of the urea headgroup mirrored that of canonical PSMA inhibitors, while the alkyl tail extended along the channel and packed against hydrophobic surfaces. Collectively, these data indicated that stearate modification neither competed with key donors/acceptors in the active site nor introduced steric clashes, and therefore did not diminish the PSMA-targeting capability of the PDC. Instead, PDC-C18 simultaneously ensured robust HSA binding and efficient PSMA recognition, offering a dual advantage for precision drug delivery. Overall, these results established stearic acid acylation as an effective modification strategy that enhanced PDC circulation stability while preserving tumor-targeting specificity, thereby laying a strong foundation for the development of long-acting PDCs.

### Mechanistic insights into PDC-C18 binding with HSA

**Fig. 3:**
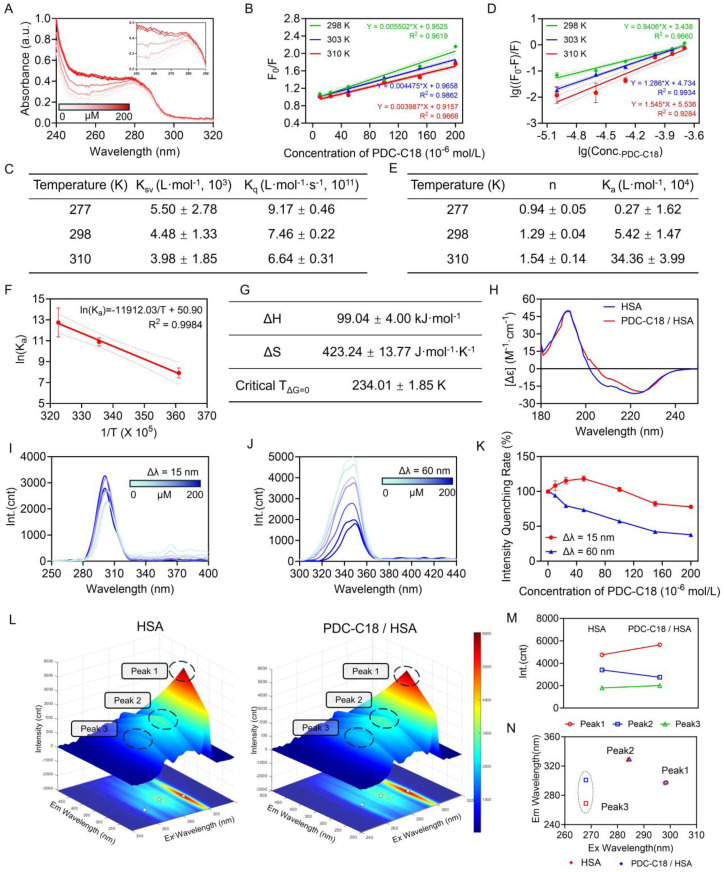
Mechanistic insights into PDC-C18 binding with HSA. (A) UV-Vis absorption spectra of HSA upon binding with PDC-C18. (B) Stern–Volmer plots of HSA fluorescence quenching by PDC-C18 at different temperatures. (C) The calculated Stern–Volmer constant (*K*_SV_) and the static quenching constant (*K*_q_) between HSA and PDC-C18 at different temperatures. (D) Double-logarithmic plots of PDC-C18–HSA binding at different temperatures and (E) corresponding binding ratios (*n*) and association constants (*K*_a_). (F) Van't Hoff plot and (G) thermodynamic parameters for the binding of PDC-C18 to HSA. (H) CD spectra of HSA before and after binding with PDC-C18. Synchronous fluorescence spectra of HSA at (I) Δ*λ* = 15 nm and (J) Δ*λ* = 60 nm and (K) corresponding fluorescence quenching rates. (L) Three-dimensional fluorescence spectra of HSA before and after binding with PDC-C18. Fluorescence peak position maps of native (M) HSA and (N) the PDC-C18/HSA complex. Data are presented as mean ± standard deviation (SD).

To elucidate the molecular basis underlying the strong binding affinity between PDC-C18 and HSA, a series of spectroscopic and thermodynamic studies were conducted. As shown in Fig. 3A, the UV absorption of HSA progressively increased with rising concentrations of PDC-C18, suggesting that ligand binding induced local conformational perturbations in HSA, thereby enhancing its UV absorption. In parallel, fluorescence spectroscopy revealed a gradual decrease in the intrinsic fluorescence intensity of HSA upon PDC-C18 titration, indicative of a quenching process (Fig. 3B and C). Stern–Volmer analysis demonstrated that the Stern–Volmer constant (*K*_SV_) decreased with increasing temperature, while the calculated static quenching constant (*K*_q_) exceeded the diffusion-controlled limit (2 × 10^10^ L mol^−1^ s^−1^). These results confirmed that quenching arose predominantly from static complex formation rather than dynamic collisions.

Further binding analysis using the double-logarithmic method revealed a binding ratio (*n*) of approximately 1.29 and 1.54 at room and physiological temperatures, respectively, pointing toward a cooperative binding effect (Fig. 3D and E). Notably, the equilibrium association constant (*K*_a_) increased with rising temperature, from 0.27 × 10^4^ L mol^−1^ at 277 K to 1.54 × 10^4^ L mol^−1^ at 310 K, implying that the binding was thermodynamically favorable. This conclusion was in good agreement with ITC measurements. Thermodynamic analysis using the Van't Hoff equation indicated that the free energy change (Δ*G*) remained negative above 234.01 K, based on fitted Δ*H* (99.04 kJ mol^−1^) and Δ*S* values (423.24 J mol^−1^ K^−1^). This suggested that the binding was spontaneous under 310 K (Fig. 3F and G). Meanwhile, the positive enthalpy and entropy changes suggested that hydrophobic interactions were the dominant driving force, which was consistent with molecular docking simulations and the theories of Timasheff, Ross, and Subramanian.^17^

Since protein function was intimately linked to structural integrity, we next examined whether PDC-C18 binding induced alterations in the secondary structure of HSA. Circular dichroism (CD) analysis demonstrated that the α-helix content of HSA decreased markedly from 43% to 20% upon ligand binding, reflecting substantial conformational rearrangements (Fig. 3H). Such a structural transition often correlated with the stabilization of ligands within hydrophobic cavities of HSA, which might, in turn, enhance drug stability in systemic circulation. To further probe microenvironmental changes at the residue level, synchronous fluorescence spectroscopy was employed with Δ*λ* = 15 nm and 60 nm to selectively monitor tyrosine and tryptophan residues, respectively (Fig. 3I–K). Increasing concentrations of PDC-C18 caused a stronger quenching effect on tyrosine residues, about only 37.84% at the concentration of 200 µM, accompanied by increased polarity and reduced hydrophobicity of their local environment. By contrast, tryptophan residues displayed only mild changes, with a slight rebound in fluorescence intensity at specific concentrations, suggesting reduced polarity and enhanced hydrophobicity in the tryptophan microenvironment. The stronger quenching rate for tyrosine compared with tryptophan highlighted the preferential sensitivity of tyrosine sites to PDC-C18 binding.

Three-dimensional fluorescence contour mapping provided further validation of these local perturbations. Specifically, peak 2 (*λ*_Ex_ = 284 nm, *λ*_Em_ = 329 nm), corresponding to the intrinsic fluorescence of HSA, exhibited marked intensity reduction after PDC-C18 binding, with 81.03% consistent with alterations in tyrosine and tryptophan environments (Fig. 3L–N). Moreover, a red shift in peak 3, associated with the polypeptide backbone, suggested increased flexibility or reorganization of the protein backbone. Thus, these spectral displacements provided qualitative evidence of microenvironmental remodeling induced by ligand binding. The binding of PDC-C18 to HSA was a spontaneous, thermodynamically favorable process primarily driven by hydrophobic interactions. This binding induced substantial secondary structural rearrangements, perturbed the microenvironment of aromatic residues, and remodeled the protein backbone, offering a molecular-level explanation for the strong HSA-binding capacity of PDC-C18. Such interactions might play an important role in improving its pharmacokinetic stability in systemic circulation. Additionally, as shown in Fig. S10, after binding with HSA, the particle size of PDC-C18 stabilized and decreased, with TEM images revealing that the complex formed uniform spherical nanoparticles. This further suggested that the binding of PDC-C18 with HSA not only induced structural rearrangements at the molecular level but also promoted the uniformity and stabilization of the complex. This uniform spherical structure was likely driven by hydrophobic interactions between PDC-C18 and HSA, which further enhanced the stability of the binding. This spherical structure and binding stability provided an ideal binding mode for PDC-C18, potentially enhancing its affinity and binding ability to HSA, thereby further strengthening the stability of PDC-C18 in aqueous phases, and offering more robust structural support for its subsequent biological functions and activities.

### Improved pharmacokinetic profiles and tumor targeting of PDC-C18

**Fig. 4:**
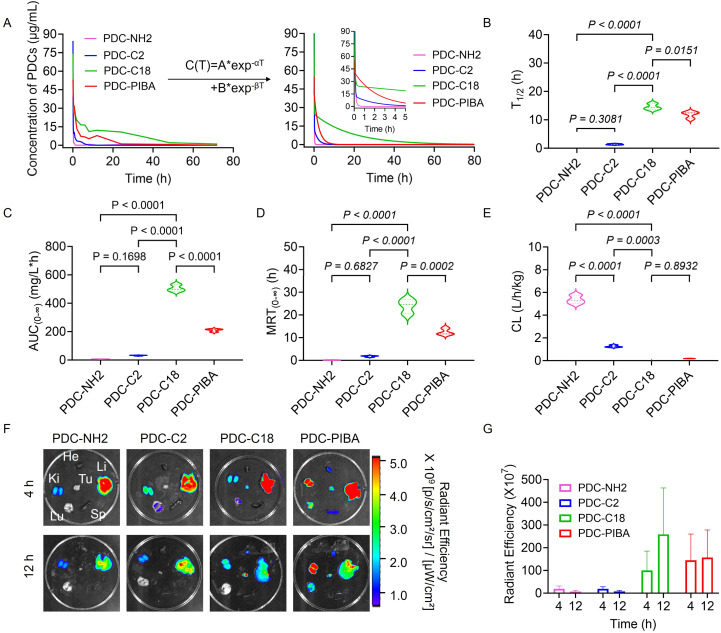
Improved pharmacokinetic profiles and tumor targeting of PDC-C18. (A) Plasma concentration–time profiles and pharmacokinetic fitting curves of the different PDCs after intravenous injection. (B) Elimination half-life (*t*_1/2_) of the PDCs. (C) Area under the plasma concentration–time curve (AUC) of the PDCs. (D) Mean residence time (MRT) of the peptides. (E) Clearance rate (CL) of the PDCs. (F) *In vivo* fluorescence imaging of tumor-bearing mice at 4 h and 12 h post-injection, showing peptide biodistribution. Tu, He, Li, Sp, Lu, and Ki represent tumor, heart, liver, spleen, lung, and kidney, respectively. (G) Quantitative analysis of fluorescence intensity in tumor tissues at 4 h and 12 h. Data in violin plots represent the full distribution of individual data points, with median indicated. Other data are presented as mean ± standard deviation (SD). Statistical significance was determined using one-way ANOVA followed by Tukey's multiple comparisons test. *P* values are indicated in the figure.

Having established the superior HSA-binding ability of PDC-C18, attention was then directed toward evaluating the pharmacokinetic behavior of the PDCs. As shown in Fig. 4A, blood concentration–time curves following intravenous administration revealed that both PDC-C18 and PDC-PIBA displayed markedly improved pharmacokinetic behaviors compared with their non-HSA-binding counterparts. This advantage stemmed from their ability to rapidly associate with HSA under 310 K (body temperature), thereby exploiting the prolonged systemic circulation of albumin. By contrast, PDC-NH_2_ and PDC-C2, which lacked HSA-binding groups, were rapidly eliminated from circulation. Notably, PDC-C18 exhibited exceptional pharmacokinetic parameters. Its half-life reached 14.6 h, representing a >160-fold improvement over the N-terminal-exposed PDC-NH_2_ (5.4 min) (Fig. 4B). The AUC (505.33 mg L^−1^ h^−1^) and MRT (23.93 h) of PDC-C18 far exceeded those of PDC-PIBA (209.67 mg L^−1^ h^−1^ and 12.47 h, respectively), demonstrating its superior circulation profile (Fig. 4C and D). In addition, PDC-C18 displayed an ultralow clearance rate (CL = 0.08 L h^−1^ kg^−1^) (Fig. 4E), a combination of features that allowed for the maintenance of therapeutic concentrations with reduced dosing frequency, which was an important consideration for improving patient compliance.

To evaluate whether these pharmacokinetic advantages translated into improved tumor targeting, *in vivo* fluorescence imaging was conducted. As shown in Fig. S11, solid tumors of prostate cancer with high PSMA expression were constructed using PC3-PIP cell line transfected with PSMA gene. As shown in Fig. 4F, PDCs without HSA-binding ability (PDC-NH_2_ and PDC-C2) produced intense fluorescence signals in the kidney and liver at both 4 h and 12 h post-injection, consistent with rapid renal and hepatic clearance. In contrast, PDC-C18 and PDC-PIBA demonstrated prominent accumulation in tumor tissues as early as 4 h, validating their enhanced tumor targeting capacity. Importantly, PDC-C18 maintained a much stronger and more sustained tumor signal at 12 h compared with PDC-PIBA, suggesting superior tumor retention and reduced off-target distribution. Quantitative fluorescence analysis further confirmed that PDC-C18 achieved the highest radiant efficiency at both 4 h and 12 h, with a time-dependent increase in tumor enrichment (Fig. 4G). By comparison, PDC-PIBA showed moderate but less durable accumulation, while PDC-NH_2_ and PDC-C2 exhibited negligible tumor localization throughout. To sum up, these results demonstrated that PDC-C18 combined prolonged circulation with selective tumor accumulation and sustained retention, hallmarks of an ideal tumor targeting therapeutic. To further investigate the independent effects of C18 and the PSMA-targeting group, we synthesized a peptide PSMA–PDC-C18 without the PSMA-targeting moiety. As shown in Fig. S12, even without the PSMA-targeting moiety, PSMA^−^PDC-C18 still exhibited an AUC of 117.82 ± 8.44 (mg L^−1^ h^−1^), a half-life of 22.51 ± 0.37 hours, and a clearance rate as low as 0.34 ± 0.02 (L h^−1^ kg^−1^), significantly outperforming PDC-NH_2_ in pharmacokinetic parameters. This result suggested that the prolonged circulation of peptide drugs was attributed to the binding of C18 to HSA. Importantly, *in vivo* fluorescence imaging further revealed a clear functional distinction between circulation and tumor targeting. While the non-PSMA peptide displayed prolonged circulation, it showed minimal tumor accumulation and retention, in contrast to the PSMA-targeted PDC-C18, which exhibited strong and sustained tumor localization. These results indicated that C18 modification governed circulation stability, whereas PSMA targeting was essential for tumor-specific accumulation and retention, highlighting the complementary but distinct roles of albumin binding and active targeting. The HSA-binding modification strategy not only extended systemic exposure but also enhanced tumor delivery, offering clear pharmacological advantages over traditional PDCs. Such properties positioned PDC-C18 as a promising candidate for further clinical translation in prostate cancer therapy.

### PDC-C18 efficiently attenuates prostate cancer progression

**Fig. 5:**
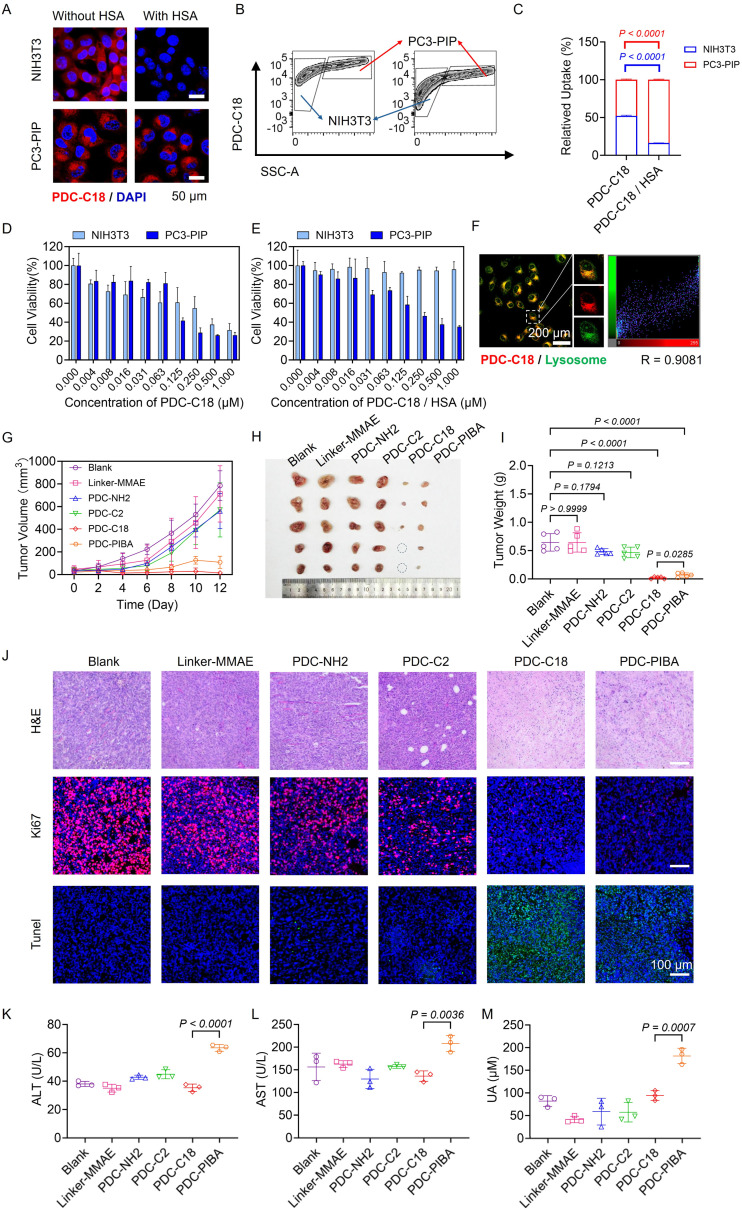
PDC-C18 efficiently inhibits prostate cancer progression. (A) Cellular uptake of PDC-C18 before and after binding to HSA in NIH3T3 and PC3-PIP cells. Scale bar: 50 µm. (B) Flow cytometry analysis and (C) relative quantification of PDC-C18 uptake in co-cultured NIH3T3 and PC3-PIP cells, with or without HSA binding (High SSC-A: PC3-PIP cells; low SSC-A: NIH3T3 cells). Cytotoxicity analysis of PDC-C18 (D) with or (E) without HSA binding in NIH3T3 and PC3-PIP cells. (F) Lysosomal localization of PDC-C18 in PC3-PIP cells (red: PDC-C18; green: lysosomes). Scale bar: 200 µm. *R* indicates Pearson's colocalization coefficient. (G) Tumor growth curves in PC3-PIP xenograft mice treated with linker-MMAE or PDC-drug. (H) Representative tumor images and (I) mass of tumors at the study endpoint. (J) Histological analysis of tumors post-treatment *via* H&E (scale bar: 100 µm), Ki67 (red, scale bar: 100 µm), and TUNEL staining (green, scale bar: 100 µm). (K) Serum alanine aminotransferase (ALT) levels. (L) Serum aspartate aminotransferase (AST) levels. (M) Uric Acid (UA) levels. Data in violin plots represent the full distribution of individual data points, with median indicated. Other data are presented as mean ± standard deviation (SD). Statistical significance was determined using unpaired *T*-test. *P* values are indicated in the figure.

Given the potent cytotoxicity of MMAE, precise tumor targeting is essential to maximize antitumor efficacy while minimizing off-target toxicity. As shown in Fig. 5A and B, free PDC-C18 displayed little targeting specificity in either single cell culture (NIH3T3 or PC3-PIP) or mixed cell culture models. This nonspecific uptake was attributed to its hydrophobic alkyl chain, which promoted passive absorption by non-target NIH3T3 cells. Strikingly, when bound to HSA, the cellular uptake profile of PDC-C18 shifted dramatically. In the mixed cell culture model, HSA complexation resulted in preferential accumulation in PSMA-positive PC3-PIP cells, with a 5.13-fold higher uptake compared to NIH3T3 cells (Fig. 5B and C). Co-immunofluorescence staining shown in Fig. S13 was conducted to assess the spatial relationship between PDC-C18 and PSMA expression. The overlay images demonstrated a high degree of colocalization between the PDC-C18 signal and PSMA-positive regions, providing direct histological evidence that tumor accumulation was mediated by PSMA targeting rather than nonspecific retention. Mechanistically, the fatty acid chain was sequestered within the hydrophobic pocket of HSA, while the PSMA-targeting moiety remained solvent-exposed, thereby improving targeting specificity and suppressing hydrophobic alkyl chain-mediated nonspecific uptake. Consistent with these (viability of 31.70% *vs.* 26.25% at 1 µM) (Fig. 5D). Upon HSA binding, however, its toxicity toward NIH3T3 cells was almost abolished (95.98% viability), while its cytotoxic potency against PC3-PIP cells was largely preserved (35.23% viability) (Fig. 5E). Further mechanistic studies revealed that PDC-C18 was internalized *via* lysosomes, where cathepsin B cleaved the responsive linker to efficiently release MMAE, thereby driving potent tumor cell killing (Fig. 5F and S14).

The therapeutic relevance of this mechanism was validated *in vivo*. As shown in Fig. 5G and H, linker-MMAE conjugates lacking albumin binding exhibited negligible therapeutic benefit due to rapid systemic clearance. Similarly, PSMA-targeting conjugates without HSA-binding capacity (PDC-NH_2_ and PDC-C2) failed to suppress tumor growth, highlighting the inherent limitations of conventional PDCs. In contrast, both PDC-C18 and PDC-PIBA achieved significant tumor suppression, with inhibition rates of 96.91% and 89.25%, respectively (Fig. 5I). Notably, PDC-C18 outperformed PDC-PIBA, indicating the pharmacological advantage of long-chain fatty acid modification in prolonging circulation and sustaining drug action. Histological analyses further confirmed these findings. As displayed in Fig. 5J, tumors from PDC-C18-treated mice showed fragmented nuclei on H&E staining, strong TUNEL signals indicative of apoptosis, and markedly reduced Ki67 expression, corroborating its robust antitumor activity. Importantly, systemic safety evaluations revealed a key advantage of PDC-C18. Compared with PDC-C18, PDC-PIBA treatment led to 1.80-, 1.53-, and 1.93-fold increases in serum ALT, AST, and UA levels, respectively (Fig. 5K–M), indicative of hepatorenal toxicity driven by the PIBA moiety. In contrast, the long-chain fatty acid modification in PDC-C18 exhibited minimal off-target toxicity, highlighting its superior biosafety over aromatic-ring-based albumin-binding strategies. Furthermore, we investigated the dose tolerance of PDC-C18. As shown in Fig. S15, when the dose reached 50 mg kg^−1^, the major organs of the tumor-bearing mice exhibited no significant damage, indicating that the high tumor targeting capability of PDC-C18 markedly reduced nonspecific organ damage, demonstrating its favorable safety profile. These results demonstrated that PDC-C18 integrated precise PSMA targeting, efficient intracellular MMAE release, potent tumor suppression, and reduced systemic toxicity, establishing it as a highly promising PDC candidate for prostate cancer therapy.

## Conclusions

In summary, the stearic acid-modified PSMA-targeting PDC (PDC-C18) was identified as a long-acting candidate for the treatment of prostate cancer. Leveraging hydrophobic interactions, PDC-C18 formed a stable *in situ* complex with HSA, markedly prolonging systemic exposure and enhancing tumor accumulation (>160-fold longer half-life, a 1.92-fold increase in mean residence time, and a 60-fold reduction in clearance). Mechanistically, albumin engagement suppressed non-specific uptake while preserving PSMA targeting, ensuring selective and potent cytotoxicity toward PSMA-positive prostate cancer cells and yielding a tumor growth inhibition of 96.91%. Compared to the conventional PIBA modification, PDC-C18 achieved superior antitumor efficacy with reduced hepatic and renal toxicity. Overall, fatty acid-mediated albumin binding provided a robust pathway for optimizing the pharmacokinetics, safety, and therapeutic index of PDCs, and PDC-C18 exemplified a rationally designed, long-acting precision therapy for prostate cancer.

## Ethical statement

All animal procedures were performed in accordance with the Guidelines for Care and Use of Laboratory Animals of Southern Medical University and approved by the Animal Ethics Committee of Southern Medical University.

## Author contributions

Ziwen Qiu: conceptualization, investigation, data curation, writing-original draft. Xiaorui Zheng: methodology, investigation. Shoumei Pan, Yingtao Zhong, Xiayun Chen, Xuejun Wen: investigation. Xin Chen, Shiying Li, Hong Cheng: conceptualization, funding acquisition, writing-review & editing. Xiaoyuan Chen: conceptualization, project administration, supervision.

## Conflicts of interest

Xiaoyuan Chen is a co-founder of and holds shares in Yantai Lannacheng Biotechnology Co., Ltd. The other authors do not have conflicts to declare.

## Supplementary Material

SC-017-D5SC08259E-s001

## Data Availability

Essential data are fully provided in the main text and supporting information (SI). Supplementary information is available. See DOI: https://doi.org/10.1039/d5sc08259e.
