# Supplementary material for: Stearic acid-modified PSMA-targeting peptide–drug conjugate for long-acting prostate cancer therapy
Source: Chem Sci. 2026 Mar 5;17(17):8651–61. doi: 10.1039/d5sc08259e (PMC12973224; doi:10.1039/d5sc08259e)
Supplement: SC-017-D5SC08259E-s001 [file SC-017-D5SC08259E-s001.pdf]

Supplementary Information (SI) for Chemical Science.
This journal is © The Royal Society of Chemistry 2026

Supporting Information

Stearic Acid-Modified PSMA-Targeting Peptide-Drug Conjugate for Long-Acting Prostate Cancer Therapy

Ziwen Qiu‡, Xiaorui Zheng‡, Shoumei Pan, Yingtao Zhong, Xiayun Chen, Xuejun Wen, Xin Chen, Shiying Li*, Hong Cheng*, and Xiaoyuan Chen**

Experimental Section

1. Materials

Resins, amino acids, and their derivatives were purchased from GL Biochem (Shanghai, China). 4-(p-Iodophenyl)butyric acid and 18-(tert-butoxy)-18-oxooctadecanoic acid were obtained from Bidepharm (Shanghai, China). Recombinant human serum albumin was acquired from Yuanye (Shanghai, China). High-concentration basement membrane matrix (Matrigel) was from Beyotime (Shanghai, China). Cy5-NHS (ester) was purchased from Shanghai Macklin Biochemical Co., Ltd. The PSMA monoclonal antibody was obtained from Proteintech (Wuhan, China). Immunofluorescence staining and blood biochemical analysis were performed by Servicebio (Wuhan, China) and Haotianqi (Changzhou, China).

2. Chemical Synthesis and Characterization of *PDC-NH2*, *PDC-C2*, *PDC-C18*, and *PDC-PIBA*

Step 1: L-Glutamic acid di-tert-butyl ester hydrochloride (261 g/mol, 1 eq, 341 mg, ***Compound 1***) and N,N-diisopropylethylamine (DIPEA, 129.247 g/mol, 3 eq, 0.648 mL) were dissolved in dichloromethane (DCM, 30 mL). The resulting mixture was stirred in a dry ice bath for 30 minutes. Triphosgene (BTC, 296.748 g/mol, 0.34 eq, 129.28 mg) was dissolved in DCM (30 mL) and added dropwise to the cooled solution. After complete addition, the reaction mixture was warmed to room temperature and stirred for 2 hours to yield ***Compound 2***.

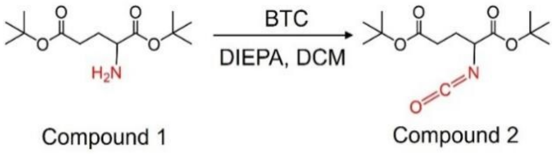

Step 2: 2-CTC resin (1.307 mmol/g, 1 g) was swollen in DCM for 30 minutes. Fmoc-Lys(Alloc)-OH (454 mg/mmol, 5 eq, 2.967 g, ***Compound 3***) was dissolved in DCM and added to the swollen resin. The mixture was reacted for 5 hours to yield ***Compound 4***. The resin was capped with a solution of methanol/DIPEA/DCM (1:2:7) and then treated with 50% piperidine (PIP) in DCM for 30 minutes. The intermediate from ***Step 1 (Compound 2)*** was immediately added to the reaction tube, followed by additional DIPEA (129.247 g/mol, 3 eq, 0.648 mL).

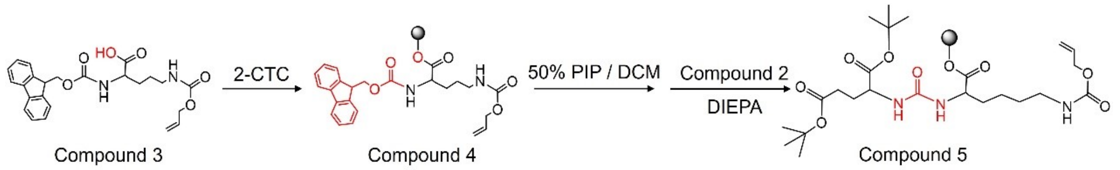

Step 3: A solution of tetrakis(triphenylphosphine)palladium(0) ($Pd(PPh_3)_4$, 1155.56 g/mol, 0.1 eq, 151 mg) and morpholine (87.122 g/mol, 10 eq, 1.127 mL) in DCM was added to ***Compound 5***. The reaction was stirred for 2 hours to remove the Alloc protecting group, yielding ***Compound 6***. Subsequently, the standard solid-pHSAe peptide synthesis (SPPS) method was followed to sequentially couple Fmoc-Glu-Otbu, Fmoc-Glu-Otbu, Fmoc-Cys(Trt)-OH, Fmoc-Glu-Otbu, and Fmoc-Glu-Otbu. The specific procedure involved dissolving the protected amino acid (3 eq), O-(Benzotriazol-1-yl)-N,N,N',N'-tetramethyluronium hexafluorophosphate (HBTU, 3.6 eq), and Hydroxybenzotriazole (HOBT, 3.6 eq) in DMF, followed by the dropwise addition of DIEPA (6 eq). The resulting mixture was added to the solid-pHSAe synthesis tube and reacted with the resin for 2 hours. After the reaction, the resin was thoroughly washed with DMF. The Fmoc protecting group was then removed by treating the resin with 50% PIP/DMF for 30 minutes. The resin was washed again with DMF, and the reaction solution for the next amino acid was added. This process was repeated until ***Compound 7*** was obtained.

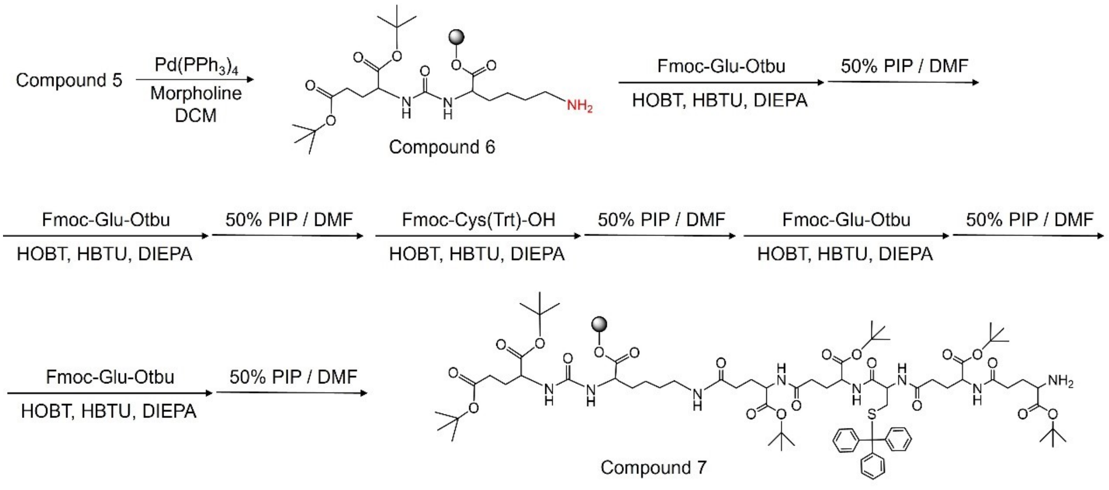

Step 4: Compound 7 was treated with a mixture of trifluoroacetic acid (TFA), anisole, 1,2-ethanedithiol (EDT), phenol, and H_2O for 5 hours. The reaction mixture was then dropwise added to ice-cold diethyl ether, and the precipitate was collected by centrifugation. Purification by HPLC yielded the N-terminal exposed PSMA-targeting peptide, ***Pep-NH2***.

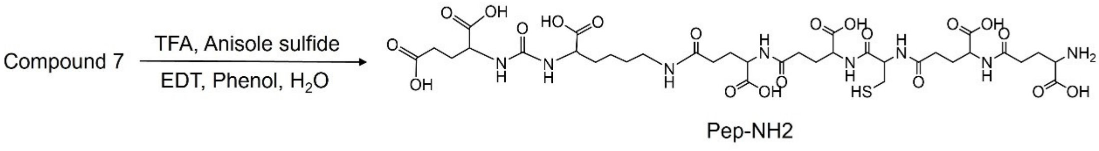

Step 5: Compound 7 was reacted with acetic anhydride/DIEPA/DMF for 2 hours. After washing with DMF and DCM, ***Step 4*** was repeated to yield the acetylated PSMA-targeting peptide, ***TPC2***.

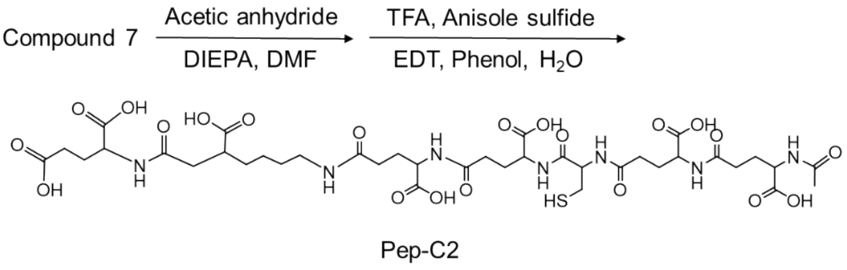

Step 6: Compound 7 was reacted with 18-(tert-butoxy)-18-oxooctadecanoic acid in the presence of DIPEA and HOBt as catalysts. ***Step 4*** was repeated to yield the stearic acid-modified PSMA-targeting peptide, ***Pep-C18***.

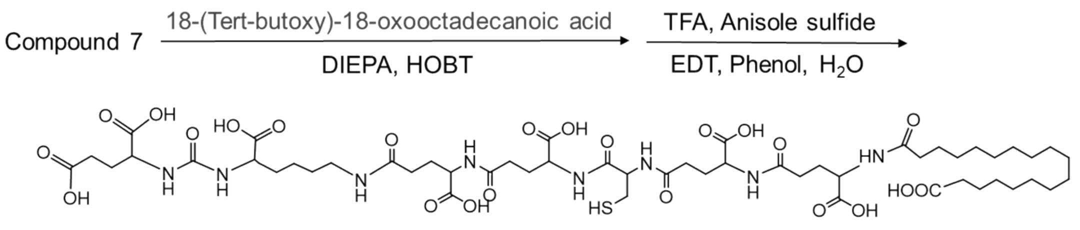

Pep-C18

Step 7: Compound 7 was reacted with 4-(p-iodophenyl)butyric acid (PIBA) in the presence of DIPEA and HOBt as catalysts. After washing with DMF and DCM, Step 4 was repeated to yield the PIBA-modified PSMA-targeting peptide, ***Pep-PIBA***.

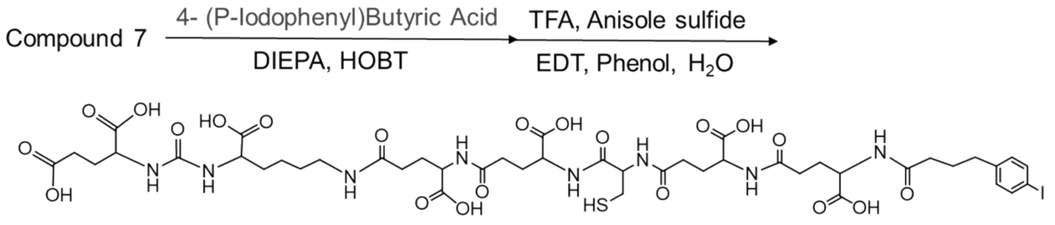

Pep-PIBA

Step 8: Maleimidocaproyl-L-valine-L-citrulline-p-aminobenzyl alcohol p-nitrophenyl carbonate (Mc-Val-Cit-PABC-PNP, 160 mg, 1 eq, ***Compound 8***), HOBt (29.3 mg, 1 eq), monomethyl auristatin E (MMAE, 208.7 mg, 1.5 eq, ***Compound 9***), and DIPEA (76 μL, 2 eq) were dissolved in DMF. The reaction was stirred under nitrogen for 48 hours, followed by dialysis and lyophilization. Purification by HPLC yielded ***Compound 10***.

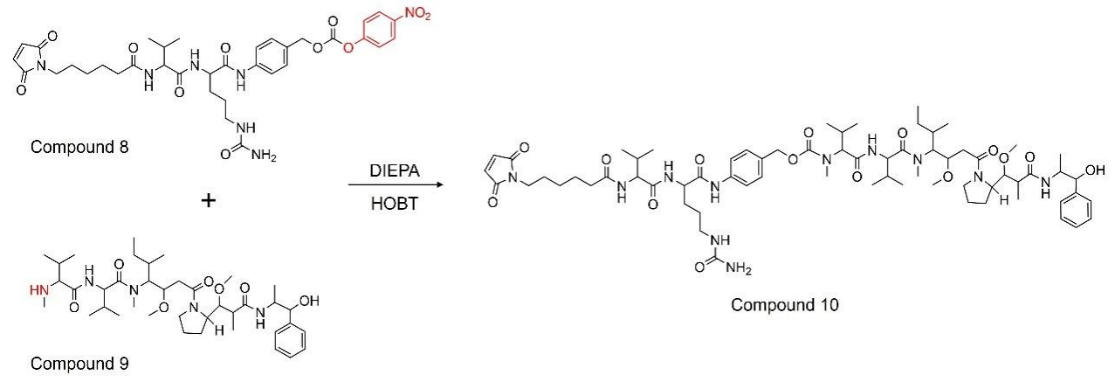

Step 9: Pep-NH$_2$ and ***Compound 10*** were dissolved in DMSO at a 1:1.05 molar ratio, with 0.05 eq DIPEA as a catalyst. The reaction was stirred under nitrogen for 48 hours, followed by dialysis and lyophilization to yield the PSMA-targeting peptide with an exposed amino group conjugated to MMAE *via* a linker, named ***PDC-NH$_2$***.

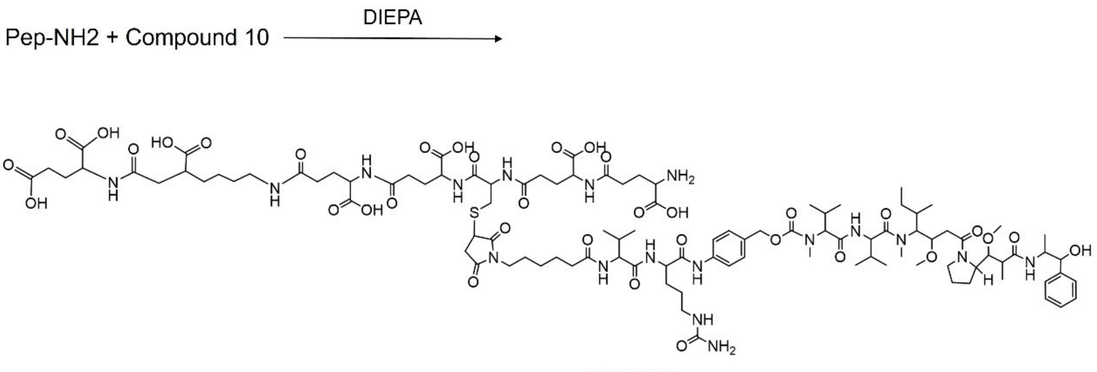

PDC-NH2

Step 10: Pep-C2 and ***Compound 10*** were dissolved in DMSO at a 1:1.05 molar ratio, with 0.05 eq DIPEA as a catalyst. The reaction was stirred under nitrogen for 48 hours, followed by dialysis and lyophilization to yield the acetylated PSMA-targeting peptide conjugated to MMAE *via* a linker, named ***PDC-C2***.

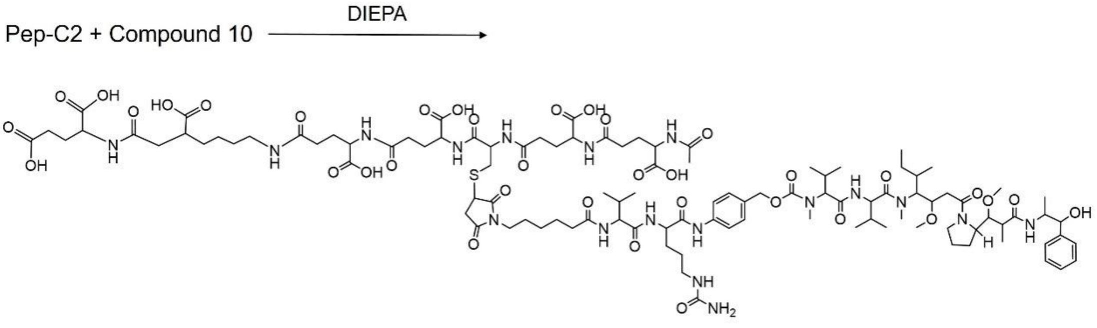

PDC-C2

Step 11: Pep-C18 and ***Compound 10*** were dissolved in DMSO at a 1:1.05 molar ratio, with 0.05 eq DIPEA as a catalyst. The reaction was stirred under nitrogen for 48 hours, followed by dialysis and lyophilization to yield the stearic acid-modified PSMA-targeting peptide conjugated to MMAE *via* a linker, named ***PDC-C18***.

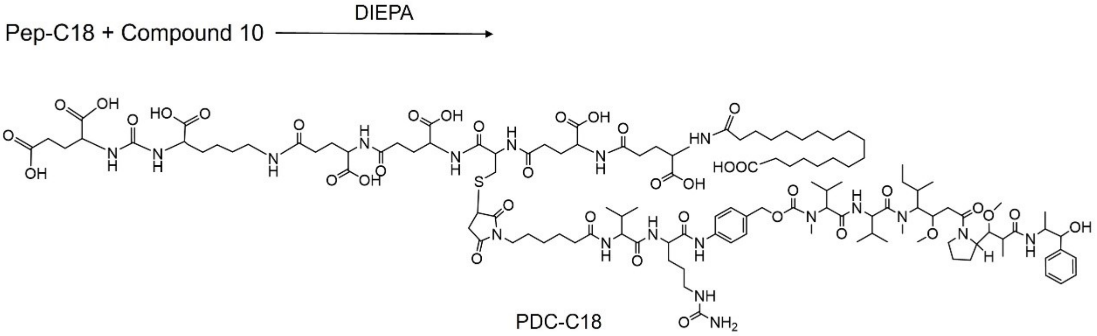

PDC-C18

Step 12: Pep-PIBA and ***Compound 10*** were dissolved in DMSO at a 1:1.05 molar ratio, with 0.05 eq DIPEA as a catalyst. The reaction was stirred under nitrogen for 48 hours, followed by dialysis and lyophilization to yield the PIBA-modified PSMA-targeting peptide conjugated to MMAE *via* a linker, named ***PDC-PIBA***.

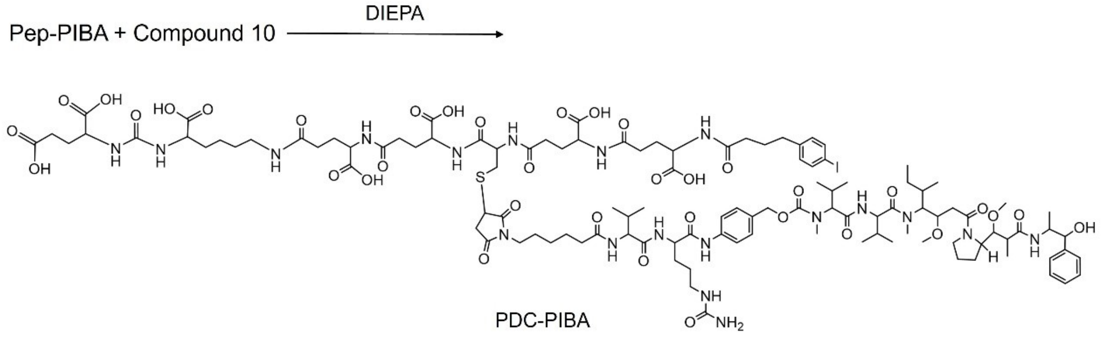

Step 13: The molecular weights of ***Pep-NH$_2$, Pep-C2, Pep-C18, Pep-PIBA, PDC-NH$_2$, PDC-C2, PDC-C18,*** and ***PDC-PIBA*** were characterized by LC-MS/MS to confirm the correctness of the synthesis.

3. Molecular Docking

Firstly, the molecular structures of PDC-NH$_2$, PDC-C2, PDC-C18, and PDC-PIBA were drawn using ChemDraw and then subjected to energy minimization with Chem3D, employing the MM94 force field to obtain the most stable conformations. The three-dimensional structure of human serum albumin (HSA) (PDB ID: 1AO6) was downloaded from the RCSB PDB database (https://www.rcsb.org) and preprocessed using Discovery Studio software by removing water molecules and adding hydrogen atoms. Subsequently, molecular docking of the small molecules with HSA was performed using the HDOCK SERVER (https://hdock.phys.hust.edu.cn/) developed by Huang's Lab. After docking, the complex with the lowest binding energy was selected, and its binding residues were analyzed using the Protein-Ligand Interaction Profiler (https://plip-tool.biotec.tu-dresden.de/plip-web/plip/index). The docking results were visualized using PyMOL. For the docking of PDC-C18 with prostate-specific membrane antigen (PSMA), the protein structure file (PDB ID: 1Z8L) was also

downloaded from the RCSB PDB database, and similar preprocessing and docking analyses were performed as described above.

4. Isothermal Titration Calorimetry (ITC)

The binding kinetics of the peptide with HSA were measured using a differential scanning calorimeter (MicroCal). Prior to the ITC experiment, HSA protein (dissolved in a glycerol solution containing 50% glycerol and 0.075 M NaCl) was placed in a dialysis bag with a molecular weight cut-off of 3 kDa. The dialysis bag was then immersed in a prepared buffer solution (20 mM Tris-HCl, pH 8.5, 130 mM NaCl) and stirred at room temperature. The dialysis buffer was changed every 24 hours for a total of three times. The final dialysis buffer was collected and supplemented with 0.05% DMSO to serve as the ITC buffer. The peptide solution was also prepared using the same ITC buffer. Due to the relatively low solubility of the peptide in the ITC buffer, reverse titration was performed to measure the binding kinetics between the protein and the peptide. Briefly, 400 μM peptide solution was placed in the ITC cell, and the syringe was filled with 10 μM HSA solution, both prepared in the same ITC buffer. A total of 20 injections were carried out, with each injection delivering 20 μL of solution at an interval of 300 seconds. The raw data obtained from the experiments were baseline-corrected and integrated using Origin 7.0 (MicroCal), with control titrations (injection of buffer into buffer) serving as references. Finally, the binding kinetics parameters were calculated and fitted using a single-site binding model.

5. Determination of the Lipid-Water Distribution Coefficient (logD)

The logD value of the peptide was determined using the n-octanol/water partition method to evaluate the distribution ability of the compound between aqueous and organic phases. First, equal volumes of n-octanol and phosphate-buffered saline (PBS, pH 7.4) were pre-mixed, thoroughly shaken to equilibrium, and then allowed to stand. The two phases were separated

using a separatory funnel and reserved for further use. The peptide was fully dissolved in PBS to prepare a stock solution at a concentration of 50 μM. Then, 1 mL of the stock solution was added to the pre-equilibrated n-octanol and aqueous phases (mixed system) and vigorously shaken for 30 minutes to achieve partition equilibrium between the two phases. After shaking, the mixture was centrifuged at 3000 rpm for 10 minutes to ensure complete phase separation. The aqueous and n-octanol phases were carefully collected, and the supernatants were analyzed quantitatively using high-performance liquid chromatography (HPLC). The distribution coefficient (D) was calculated using the following equation:

$$D = \frac{C_{octanol}}{C_{Water}}$$

where $C_{octanol}$ and C_{water} represent the concentrations of the peptide in the n-octanol and aqueous phases, respectively. Finally, the logD value was expressed as:

$$logD = log_{10}(D)$$

6. UV-Vis Absorption Spectroscopy and Circular Dichroism (CD) Analysis

PDC-C18 was fully dissolved in PBS solution and then diluted to prepare a series of gradient concentrations of secondary solutions. Subsequently, an equal volume of HSA solution was added to each sample to ensure a final protein concentration of 20 μM. The UV-Vis absorption spectra of the mixtures containing different concentrations of PDC-C18 and HSA were measured using a spectrophotometer to observe changes upon binding. In addition, CD spectra were recorded for HSA before and after binding with PDC-C18 to analyze conformational changes. The CD data were further processed using CDNN software to evaluate the alterations in the secondary structure.

7. Fluorescence Quenching Experiments and Thermodynamic Analysis

To investigate the interaction between the peptide PDC-C18 and HSA, fluorescence quenching experiments were performed. First, HSA was dissolved in PBS (pH 7.4) to prepare a working solution at a concentration of 100 μM. PDC-C18 was dissolved in the same buffer to prepare a stock solution at a concentration of 200 μM and then gradually diluted to different concentrations as needed. In the experiments, an appropriate volume of HSA solution was mixed with PDC-C18 solutions at various concentration gradients. After each addition, PBS buffer was added to adjust the total volume to the same level, ensuring a final HSA concentration of 20 μM and PDC-C18 concentrations of 10, 25, 50, 100, 150, and 200 μM, respectively. After thorough mixing, the mixtures were incubated at 277 K, 298 K, and 310 K for 60 minutes to reach binding equilibrium. Fluorescence measurements were performed using a fluorescence spectrophotometer with an excitation wavelength set at 280 nm and an emission wavelength scanning range of 300–500 nm. Both the excitation and emission slit widths were set to 5 nm, and the scanning speed was 240 nm/min. The experiments were carried out at different temperatures (277 K, 298 K, and 310 K) to investigate the temperature dependence of the binding behavior. The quenching efficiency was calculated, and Stern-Volmer plots were constructed. The standard Stern-Volmer equation is as follows:

$$\frac{F_0}{F} = 1 + K_{sv}[Q]$$

where F_0 represents the fluorescence intensity of HSA in the absence of the peptide, F is the fluorescence intensity in the presence of the peptide, Ksv is the Stern-Volmer constant reflecting quenching efficiency (unit: $L\cdot mol^{-1}$), and $[Q]$ is the peptide concentration. Furthermore, based on the static quenching model, the binding constant (K_a) and the number of binding sites (n) were calculated using the double-logarithmic equation:

$$\log\left(\frac{F_0 - F}{F}\right) = \log k_a + n\log[Q]$$

Subsequently, the Van't Hoff equation was fitted based on the binding constants (K_a) obtained at different temperatures, using the following equation:

$$\ln K_a = -\frac{\Delta H}{RT} + \frac{\Delta S}{R}$$

where R is the universal gas constant (8.314 J·mol^{-1}·K^{-1}), ΔH represents the enthalpy change, and ΔS represents the entropy change. The values of ΔH and ΔS were calculated from the slope and intercept of the fitted line, and the Gibbs free energy change (ΔG) was further determined using the equation:

$$\Delta G = \Delta H - T\Delta S = -RT\ln K_a$$

The fitting and parameter calculations were performed using Matlab R2019, and the plots were generated using GraphPad Prism (v9.5).

8. Synchronous Fluorescence Spectroscopy

To further investigate the effects of PDC-C18 binding on the microenvironment of HSA, synchronous fluorescence spectroscopy was performed. First, 20 μM HSA solution was prepared and mixed with different concentrations of PDC-C18 (e.g., 0, 10, 25, 50, 100, 150, 200 μM). The mixtures were adjusted to the same final volume with PBS buffer, thoroughly mixed, and incubated at 25°C for 60 minutes to reach binding equilibrium. Synchronous fluorescence measurements were conducted using a fluorescence spectrophotometer. The wavelength interval (Δλ) between excitation and emission was set at 15 nm and 60 nm to monitor the microenvironmental changes of tyrosine and tryptophan residues, respectively. The scanning range was set from 250 to 350 nm for both Δλ = 15 nm and Δλ = 60 nm. The excitation and emission slit widths were both set at 5 nm, and the scanning speed was 240 nm/min. The synchronous fluorescence spectra were recorded for each sample, and shifts or changes in peak intensity were analyzed to assess conformational alterations in the protein.

9. Three-Dimensional Fluorescence Spectroscopy

To further explore the overall conformational changes in HSA upon binding with PDC-C18, three-dimensional fluorescence spectroscopy was carried out. A 20 μM HSA solution was mixed with different concentrations of 200 μM PDC-C18, adjusted to the same final volume with PBS buffer, thoroughly mixed, and incubated at 37°C for 60 minutes to ensure binding equilibrium. Three-dimensional fluorescence spectra were recorded using a fluorescence spectrophotometer. The three-dimensional fluorescence spectra and corresponding contour maps were obtained, and variations in fluorescence peak positions and intensities before and after binding were analyzed to evaluate the overall conformational and microenvironmental changes in HSA.

10. *In Vivo* Pharmacokinetic Study of Peptides

To investigate the pharmacokinetic characteristics of the four Cy5.5-labeled delivery peptides (without drug payload), the peptides were conjugated with Cy5.5-NHS ester to replace the original MMAE-Linker, yielding Cy5.5-labeled peptide probes. The purified labeled peptides were dissolved in PBS buffer (pH 7.4) and prepared at a working concentration of 50 μM. The Cy5.5-labeled peptides were administered *via* a single-dose intravenous injection at a concentration of 50 μM through the tail vein. The concentration here was significantly higher than the therapeutic injection dose, primarily aimed at adequately demonstrating the tissue distribution of the peptide carrier, and was not intended to serve as a reference for the therapeutic dose. Blood samples were collected at various time intervals after administration, allowed to clot, and then centrifuged at 3000 rpm for 10 minutes to obtain the serum supernatant. The fluorescence intensity of the serum samples was measured using a fluorescence spectrophotometer to estimate the relative concentration of peptides in plasma. The resulting plasma concentration–time data were analyzed using Phoenix software, and pharmacokinetic parameters such as half-life, Area under the Curve (AUC), Mean Residence

Time (MRT) and clearance (Cl) of distribution were calculated using a single-dose two-compartment model.

11. Cellular Uptake, Toxicity Analysis, and Lysosomal Localization

NIH3T3 cells were cultured in DMEM medium supplemented with 10% FBS. The PSMA-positive cell line, designated as PC3-PIP, was established by stably transfecting PSMA-negative PC3 cells with the PSMA gene (FOLH1) followed by screening for high-expressing monoclonal clones, and maintained in F12K medium containing 10% FBS. For cellular uptake studies, Cy5.5-labeled peptides (10 μM) were pre-incubated with 20 μM HSA to form peptide-HSA complexes. The peptide alone or peptide-HSA complexes were then co-incubated with either PC3-PIP or NIH3T3 cells for 2 hours, followed by observation using confocal laser scanning microscopy (CLSM). In a separate experiment, PC3-PIP and NIH3T3 cells were mixed at a 1:1 ratio and treated with Cy5.5-labeled peptide or peptide-HSA complexes for 2 h. The cellular uptake of the PDC was analyzed by flow cytometry, with relative quantification based on fluorescence intensity. For toxicity analysis of PDC-C18, cells were treated with either 1 μM PDC-C18 alone or 1 μM PDC-C18 pre-complexed with 20 μM HSA at gradient dilutions for 24 h, followed by assessment of cell viability using the MTT assay. To evaluate lysosomal localization of PDC-C18, PC3-PIP cells were incubated with Cy5.5-labeled peptide-HSA complexes for 1 hour, followed by staining with a lysosomal probe. The intracellular distribution of the red fluorescent drug and green fluorescent lysosomes was visualized by CLSM, and the colocalization coefficient was calculated.

12. Prostate Cancer Model Establishment and Treatment

Male Babl/c-nude mice (6 weeks old) were purchased from Zhuhai Bestest. All animal procedures were performed in accordance with the Guidelines for Care and Use of Laboratory Animals of Southern Medical University and approved by the Animal Ethics Committee of

Southern Medical University. PC3-PIP cells suspended in high-concentration Matrigel were subcutaneously inoculated into the dorsal flank of the mice. Seven days post-inoculation, the mice were intravenously administered either linker-MMAE or the PDC drug (1 mg/kg) *via* the tail vein, with a second dose administered two days later. Tumor growth was monitored throughout the study. After two weeks, all mice were euthanized, and tumors were excised, photographed, and weighed. Blood samples were collected for hematological analysis. Tumor tissues were subjected to H&E staining, TUNEL assay, and Ki67 immunohistochemistry to evaluate tumor proliferation and apoptosis.

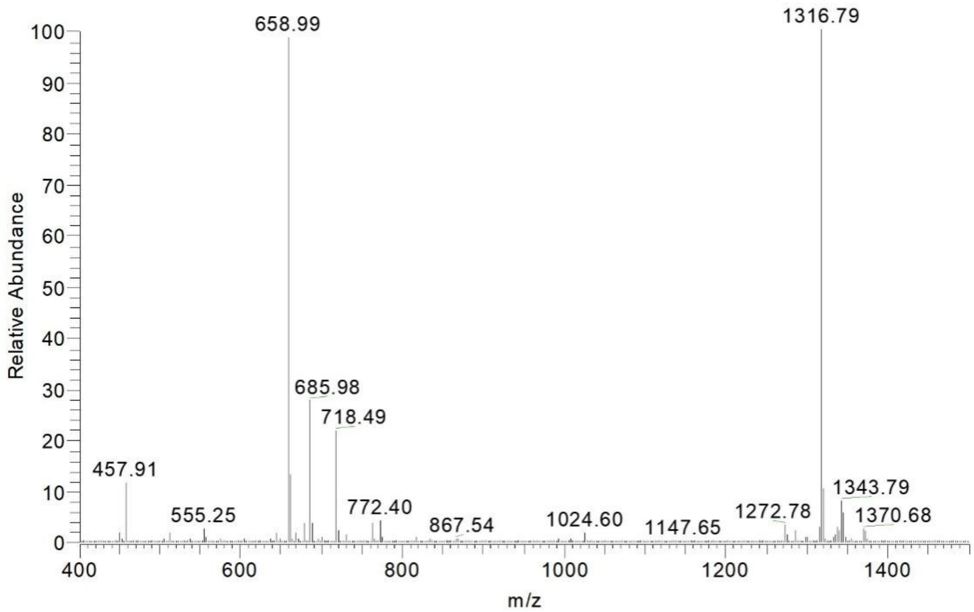

Figure S1. ESI-MS of MC-Val-Cit-PAB-MMAE. Theoretical mass: 1315.78.

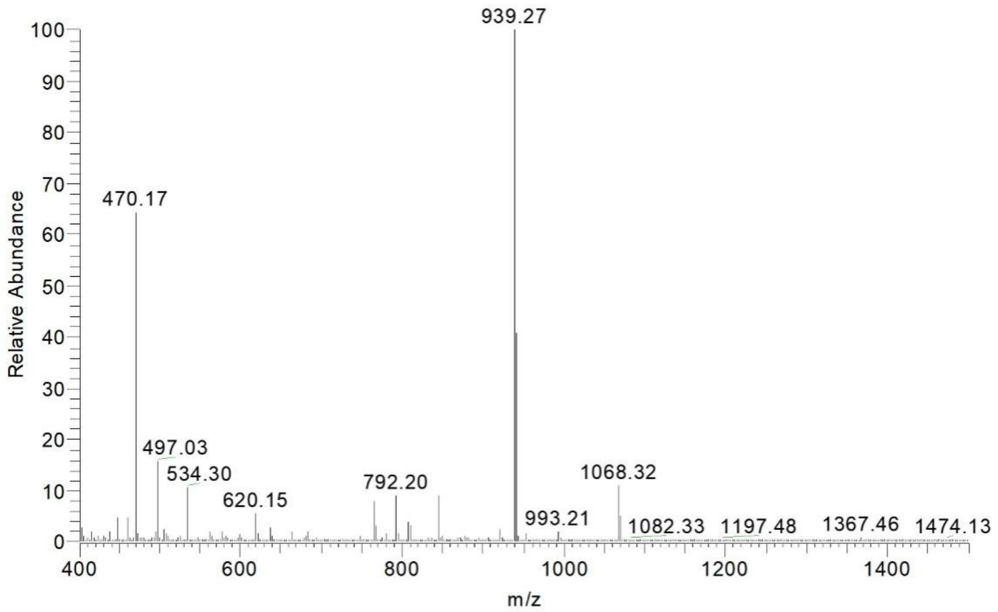

Figure S2. ESI-MS of Pep-NH_2. Theoretical mass: 938.3.

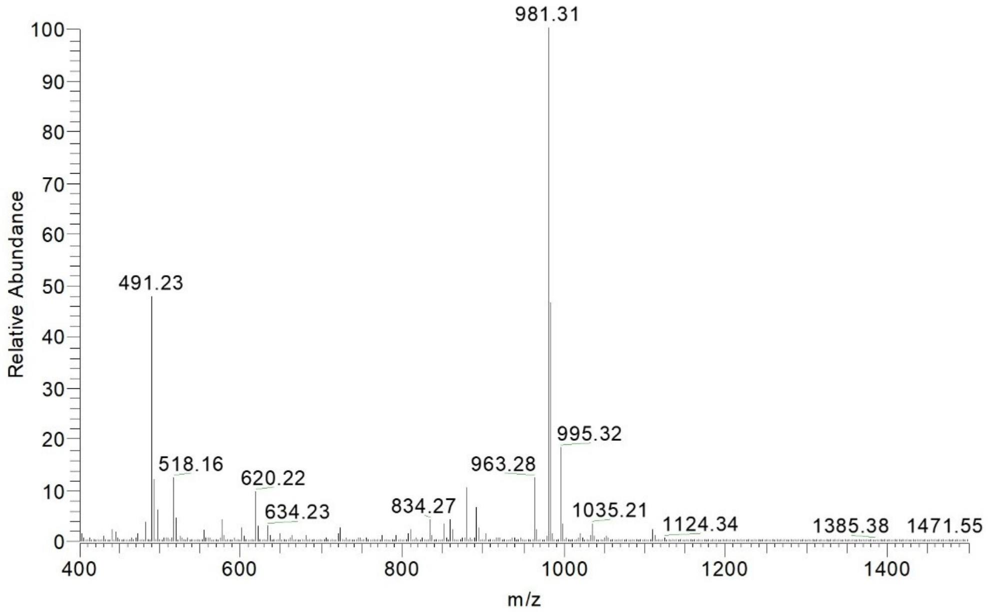

Figure S3. ESI-MS of Pep-C2. Theoretical mass: 980.3.

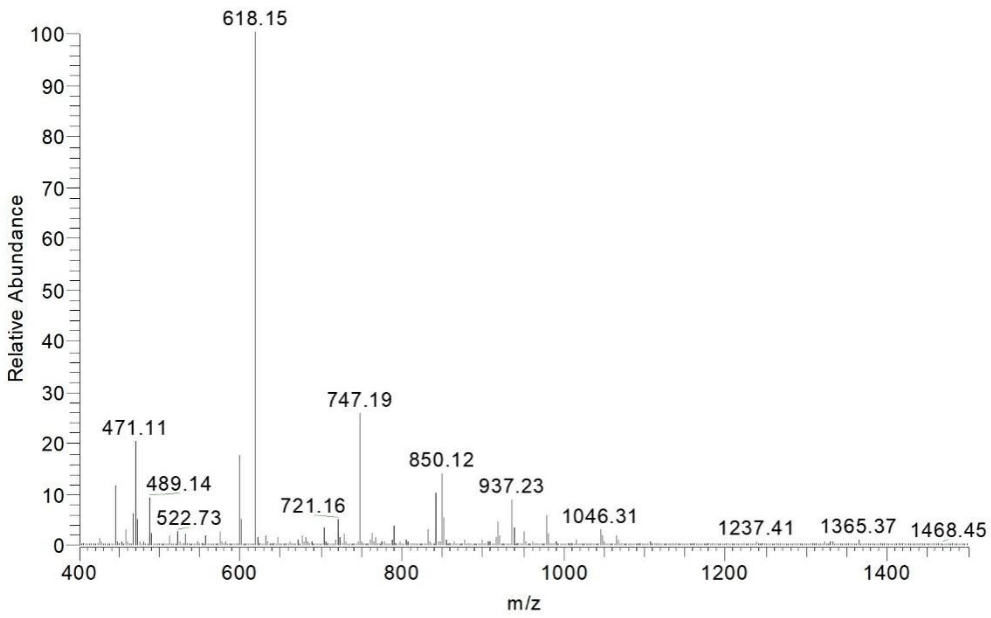

Figure S4. ESI-MS of Pep-C18. Theoretical mass: 1234.5.

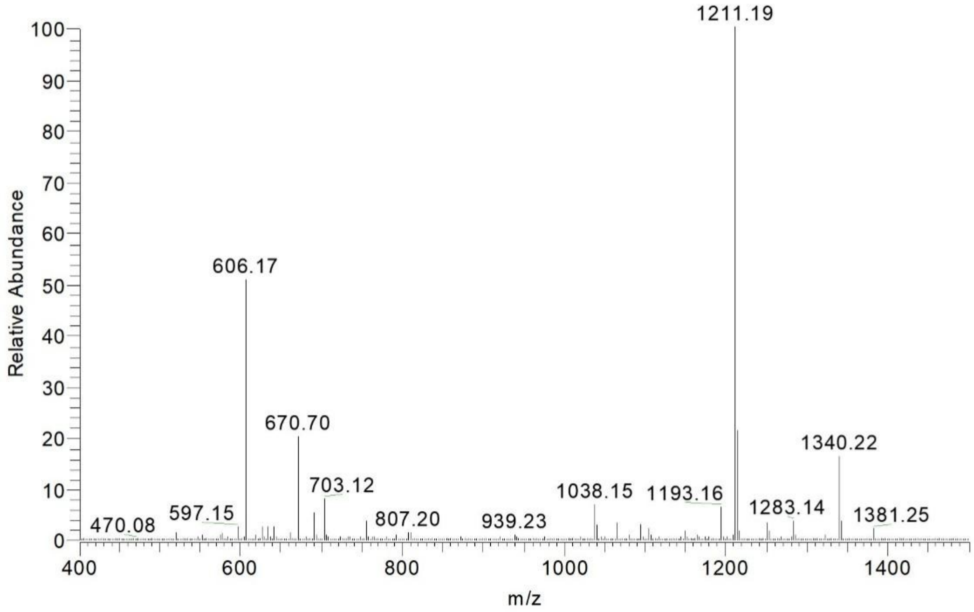

Figure S5. ESI-MS of Pep-C18. Theoretical mass: 1210.28.

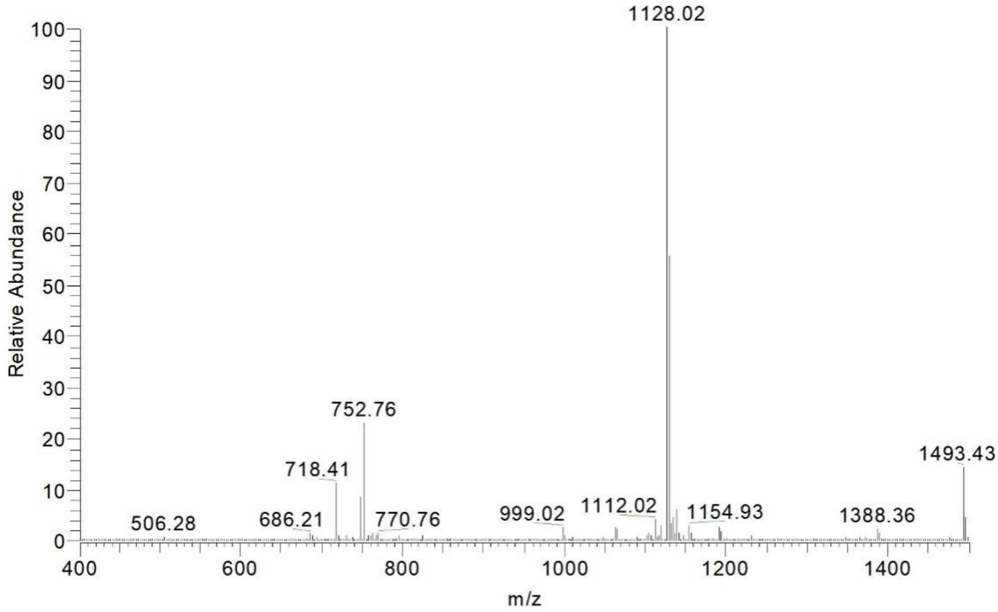

Figure S6. ESI-MS of PDC-NH2. Theoretical mass: 2255.

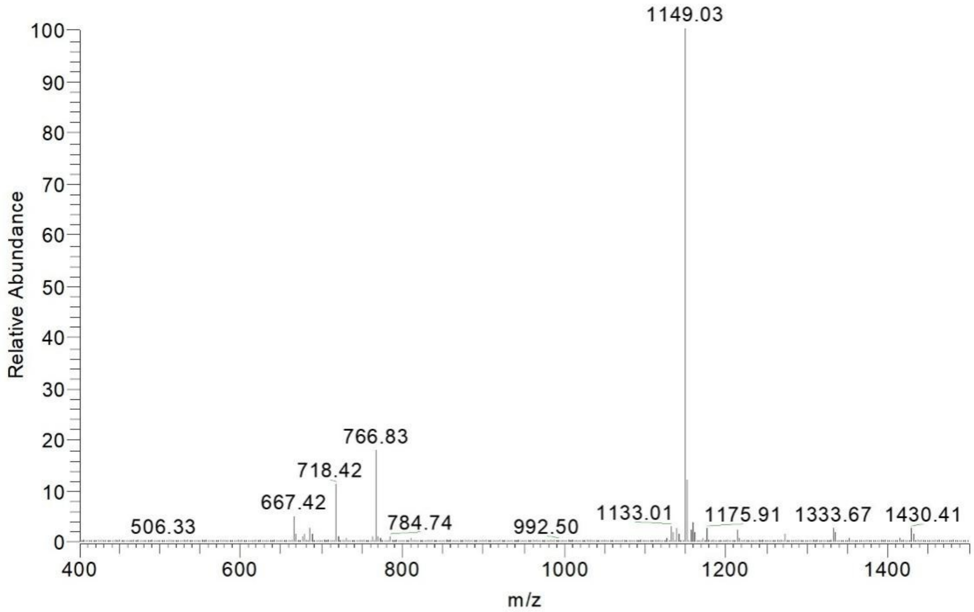

Figure S7. ESI-MS of PDC-C2. Theoretical mass: 2297.

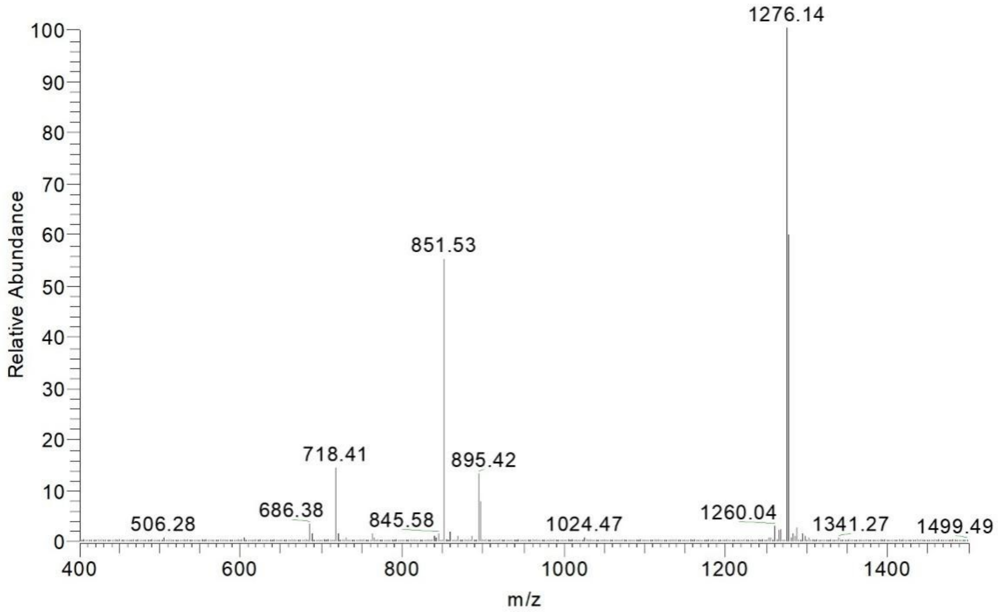

Figure S8. ESI-MS of PDC-C18. Theoretical mass: 2552.

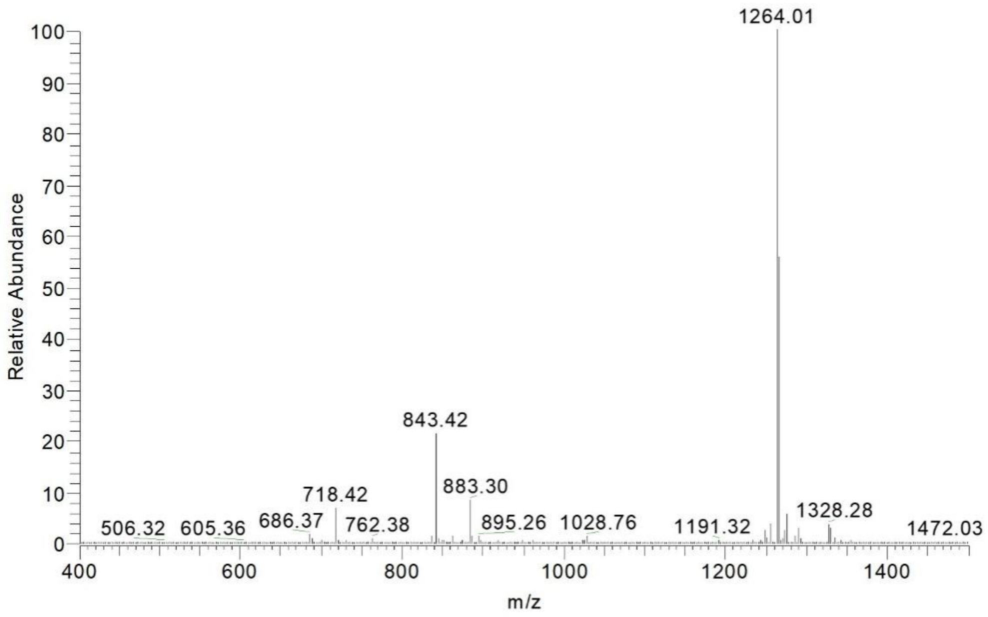

Figure S9. ESI-MS of PDC-PIBA. Theoretical mass: 2527.

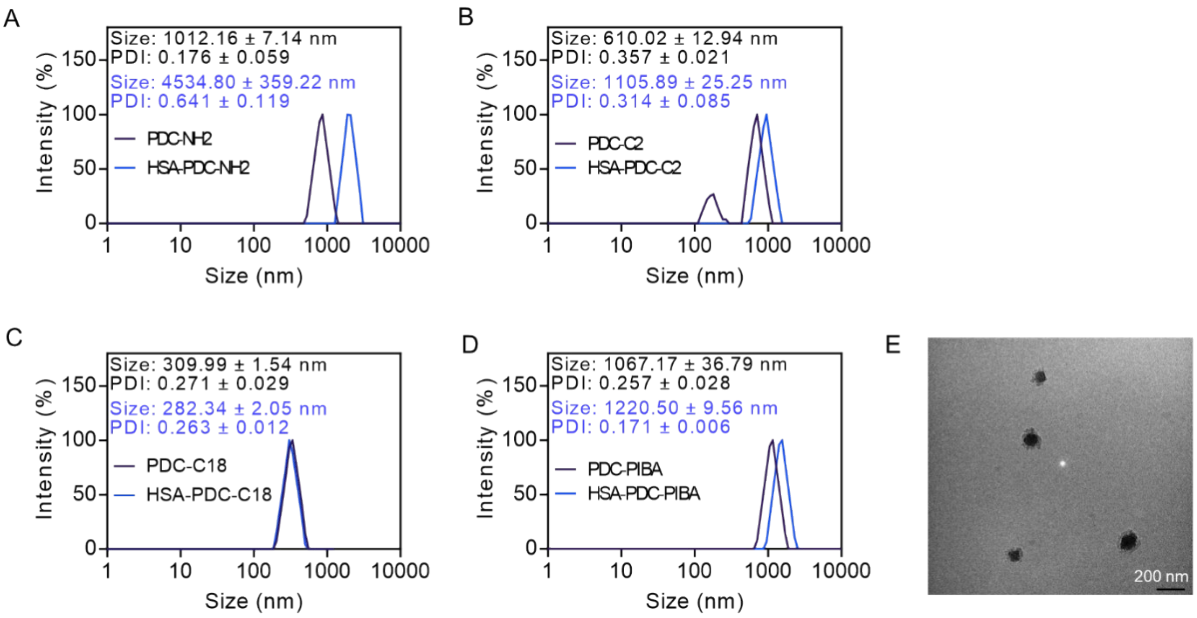

Figure S10. Particle size changes of (A) PDC-NH_2, (B) PDC-C2, (C) PDC-C18, and (D) PDC-PIBA before and after binding with HSA in aqueous solution. (E) TEM image of the HSA/PDC-C18 complex.

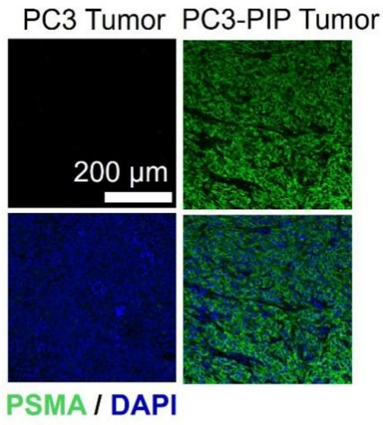

Figure S11. PSMA (green) immunofluorescence staining of PC3 tumors and PC3-PIP tumors. Scale bar: 200 µm.

A

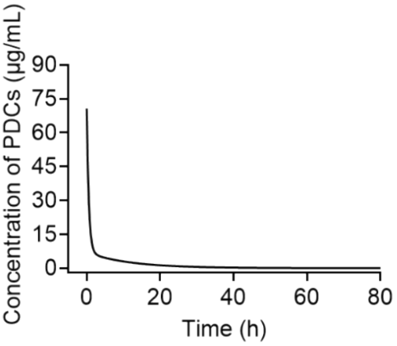

B

$T_{1/2}$ (h)	22.51 ± 0.37
$AUC_{(0-\infty)}$ (mg/L*h)	117.82 ± 8.44
$MRT_{(0-\infty)}$ (h)	8.58 ± 1.60
CL (L/h/kg)	0.34 ± 0.02

C D

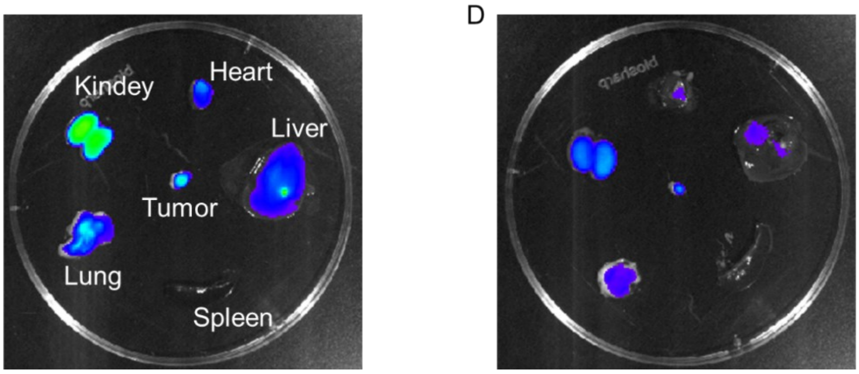

Figure S12. (A) Pharmacokinetic curve of PDC-C18 without the PSMA-targeting moiety and (B) PK analysis. After intravenous injection of PDC-C18 without the PSMA-targeting moiety, organ fluorescence distribution at (C) 4 hours and (D) 12 hours.

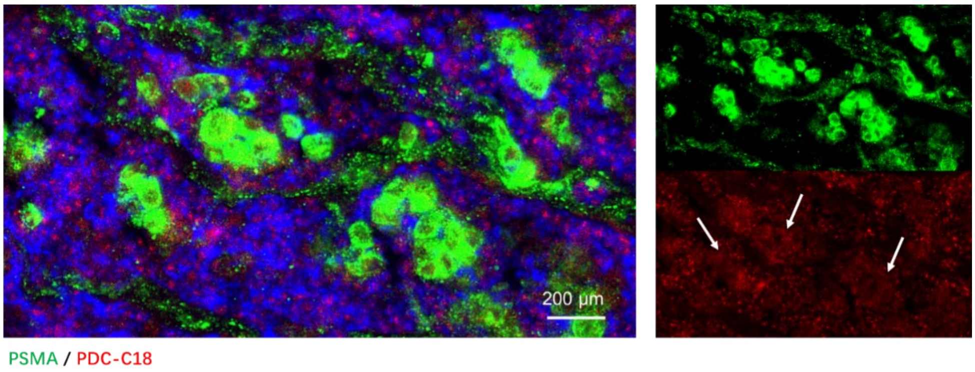

Figure S13. Localization distribution of PSMA within the tumor after injection of PDC-C18.

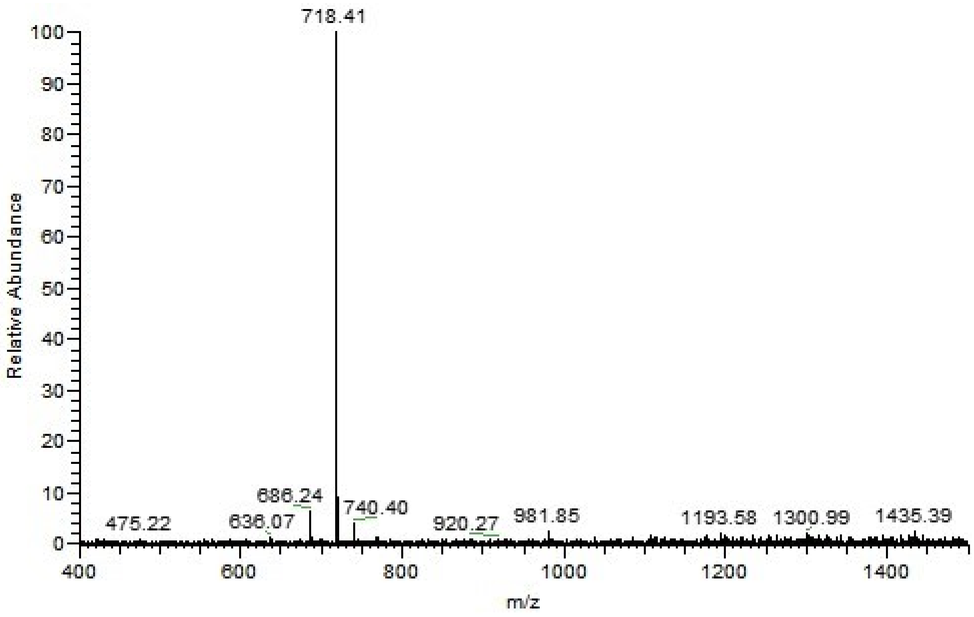

Figure S14. LC-MS/MS analysis of cathepsin B-mediated cleavage products of PDC-C18.

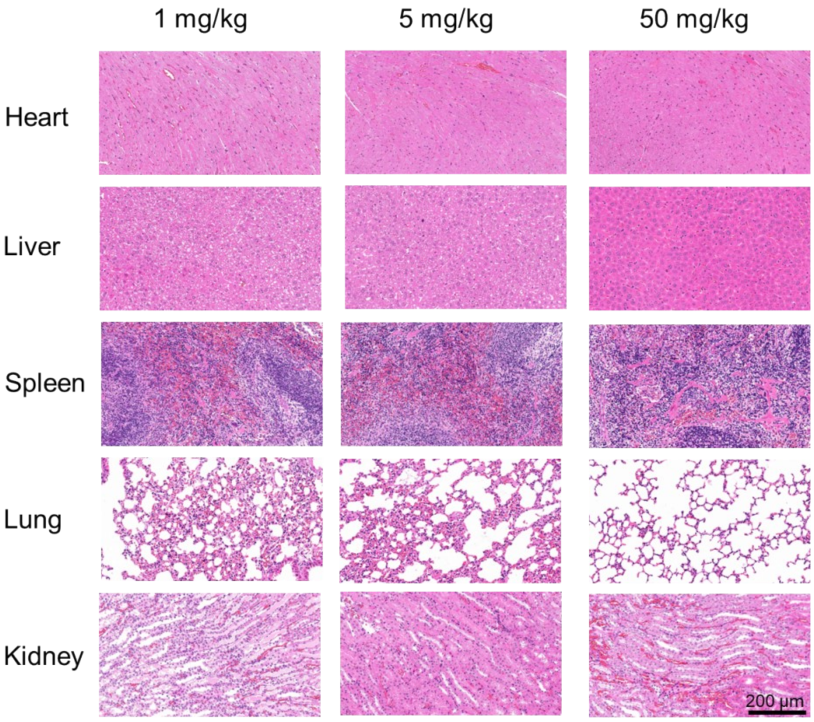

Figure S15. H&E of organs under different doses of PDC-C18. Scare bar: 200 μm.
